# Supplementary material for: Ligand binding drives proteolysis of the SmcR master transcription factor and controls quorum sensing-state transitions in Vibrio species
Source: mBio. 2026 Jan 30;17(3):e03445-25. doi: 10.1128/mbio.03445-25 (PMC12977469; doi:10.1128/mbio.03445-25)
Supplement: Supplemental figures and tables — Supplemental figures, tables, and movie legend. [file mbio.03445-25-s0001.pdf]

Ligand binding drives proteolysis of the SmcR master transcription factor and controls quorum sensing-state transitions in *Vibrio* species

Tanmaya A. Rasal, Caleb P. Mallery, Biqing Liang, Matthew W. Brockley, Chelsea A. Simpson, Abigail D. Padgett, Logan J. Geyman, Finley J. Andrew, Laura C. Brown, Jon E. Paczkowski, Julia C. van Kessel

Supplementary Information

Video S1

Tables S1-S5

Figures S1-S16

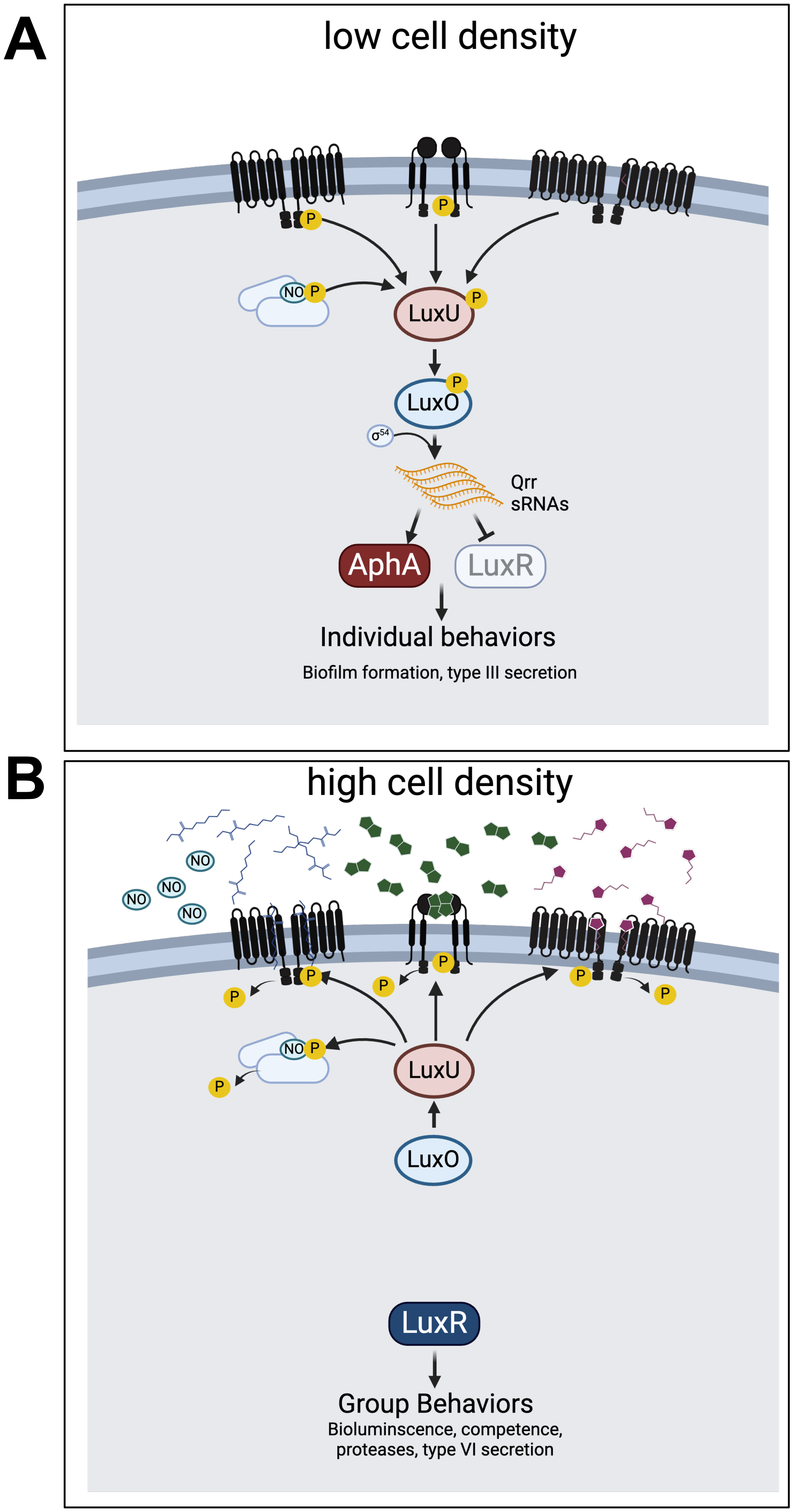

Figure S1. Current model of QS signaling at low and high cell density in *V. campbellii*. (A) At low cell density, autoinducer concentrations are low and do not bind to receptors. Receptors act as kinases to phosphorylate (P) LuxU and subsequently LuxO. Phosphorylated LuxO and Sigma-54 activate transcription of the Qrr sRNAs, which activate translation of AphA and repress translation of LuxR, leading to transcriptional regulation of genes driving individual behaviors. (B) At high cell density, high levels of autoinducers and/or nitric oxide (NO) enable binding to the receptors, which results in phosphatase activity. Dephosphorylated LuxO does not activate the Qrrs, and thus LuxR is translated to high levels. Transcription by LuxR regulates hundreds of genes required for producing group behaviors.

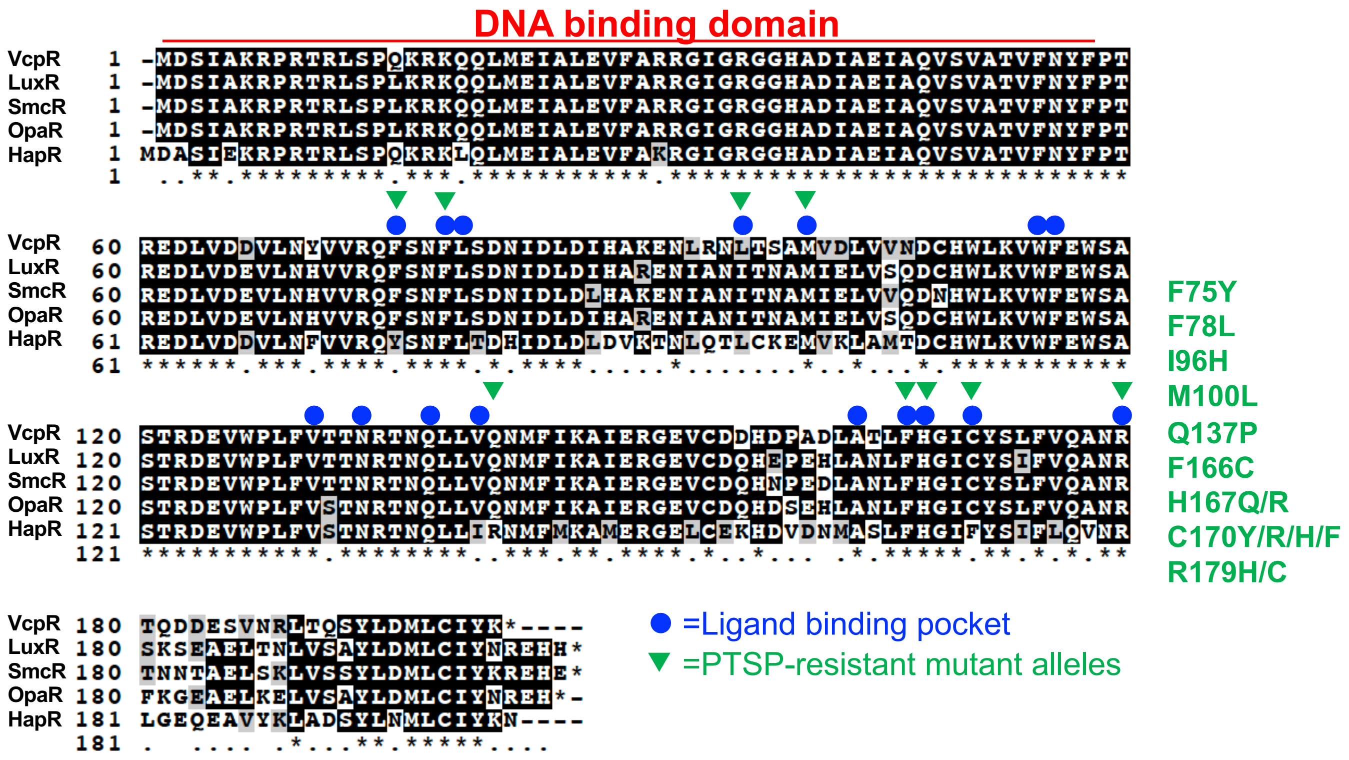

Figure S2. Alignment of the LuxR/HapR homologues. VcpR (*Vibrio coralliilyticus* OCN008), LuxR (*V. campbellii* BB120)**,** SmcR (*V. vulnificus* ATCC 27562), OpaR (*V. parahaemolyticus* RIMD2210633), and HapR (*V. cholerae* E7946). The residues identified to be part of the ligand binding pocket of SmcR-PTSP are indicated by a blue dot above the alignment position. The residues identified as PTSP-resistant alleles by genetics are indicated by green triangles above the alignment position.

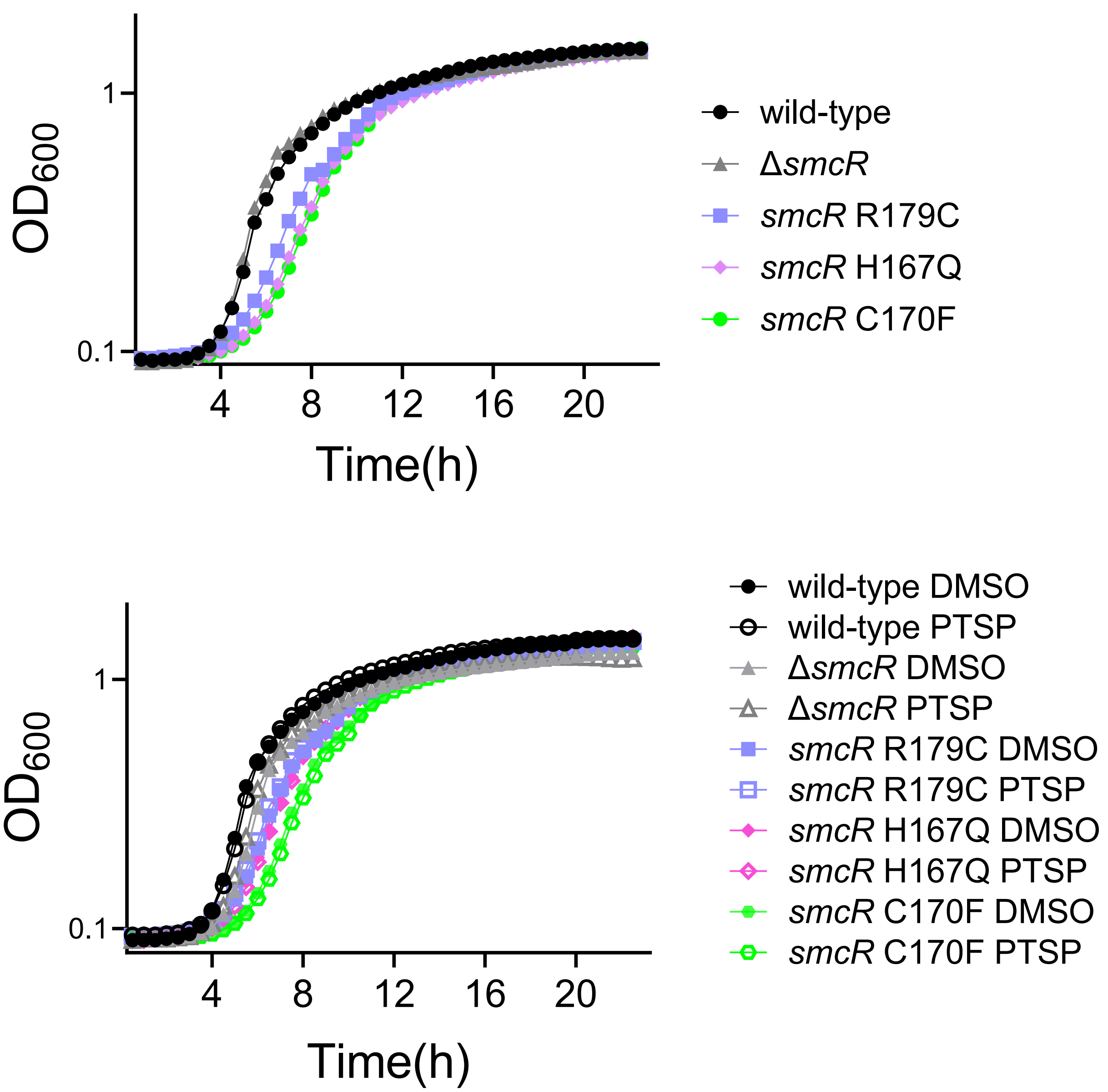

Figure S3. Addition of PTSP to *Vibrio vulnificus* does not alter growth trends. Overnight growth curve assays of SmcR protein in V*ibrio vulnificus* strains expressing wild-type *smcR,* Δ*smcR*, *smcR* R179C, H167Q, or C170F treated with 25 μM PTSP or an equivalent concentration of DMSO solvent. Figures are representative of triplicate biological assays

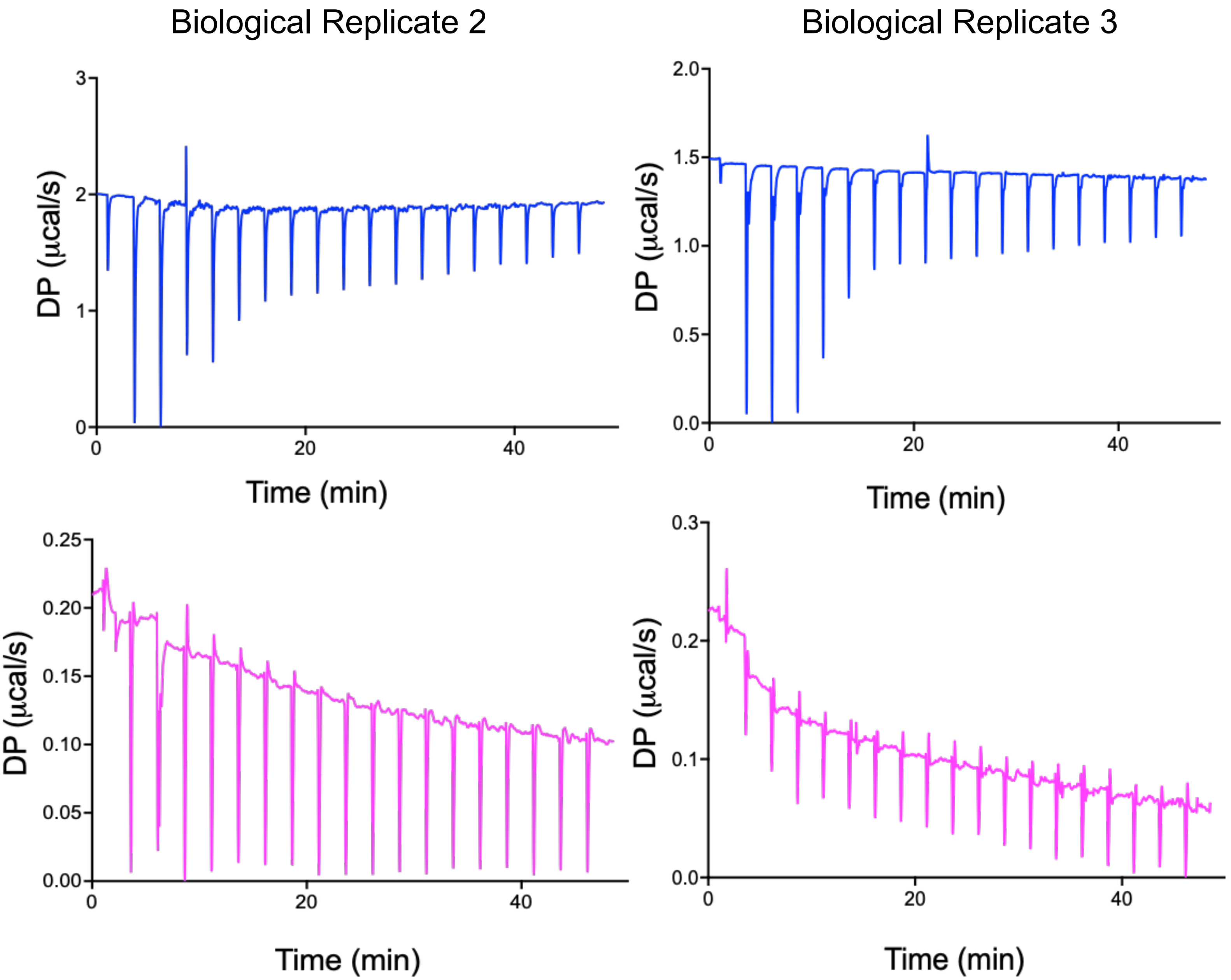

Figure S4. Isothermal titration calorimetry of wild-type SmcR and SmcR R179C. Biological replicates of isothermal titration calorimetry experiments of WT SmcR (blue) and SmcR R179C (magenta) with PTSP for Figure 1. DP denotes differential power. Different protein purification preparations were used for each replicate.

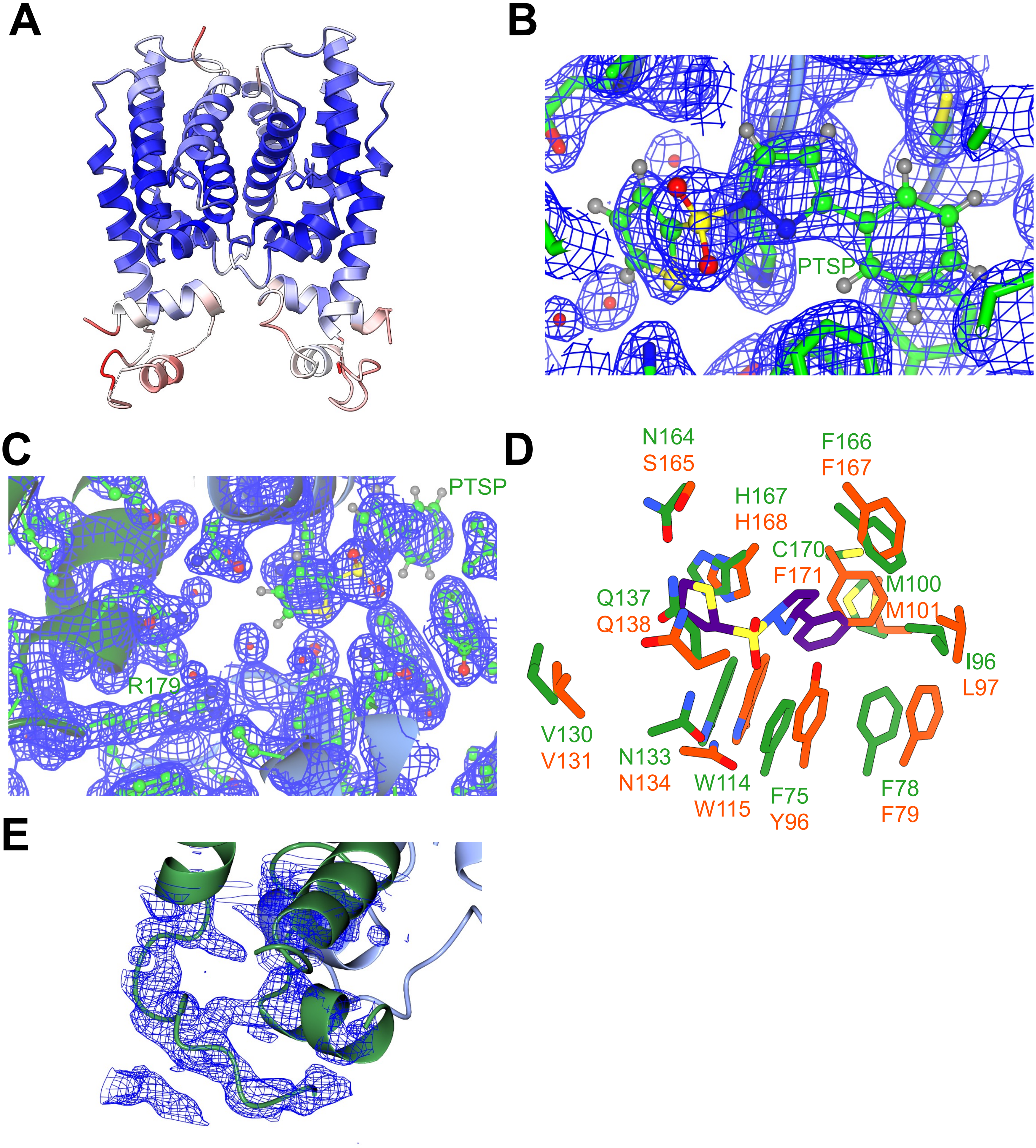

Figure S5. Validation of the SmcR-PTSP structure. (A) Structure of SmcR-PTSP colored by B-factor. Blue indicates low B-factor and red indicates high B-factor scores. (B) Example close-up view of map density of the SmcR LBP-SmcR interface and (C) R179. (D) Structural overlay of the residues important for ligand binding in SmcR (green) and HapR (orange) in their respective binding pocket relative to the orientation of PTSP (purple) in SmcR. (E) Example close-up view of map density of the SmcR N-terminal domain.

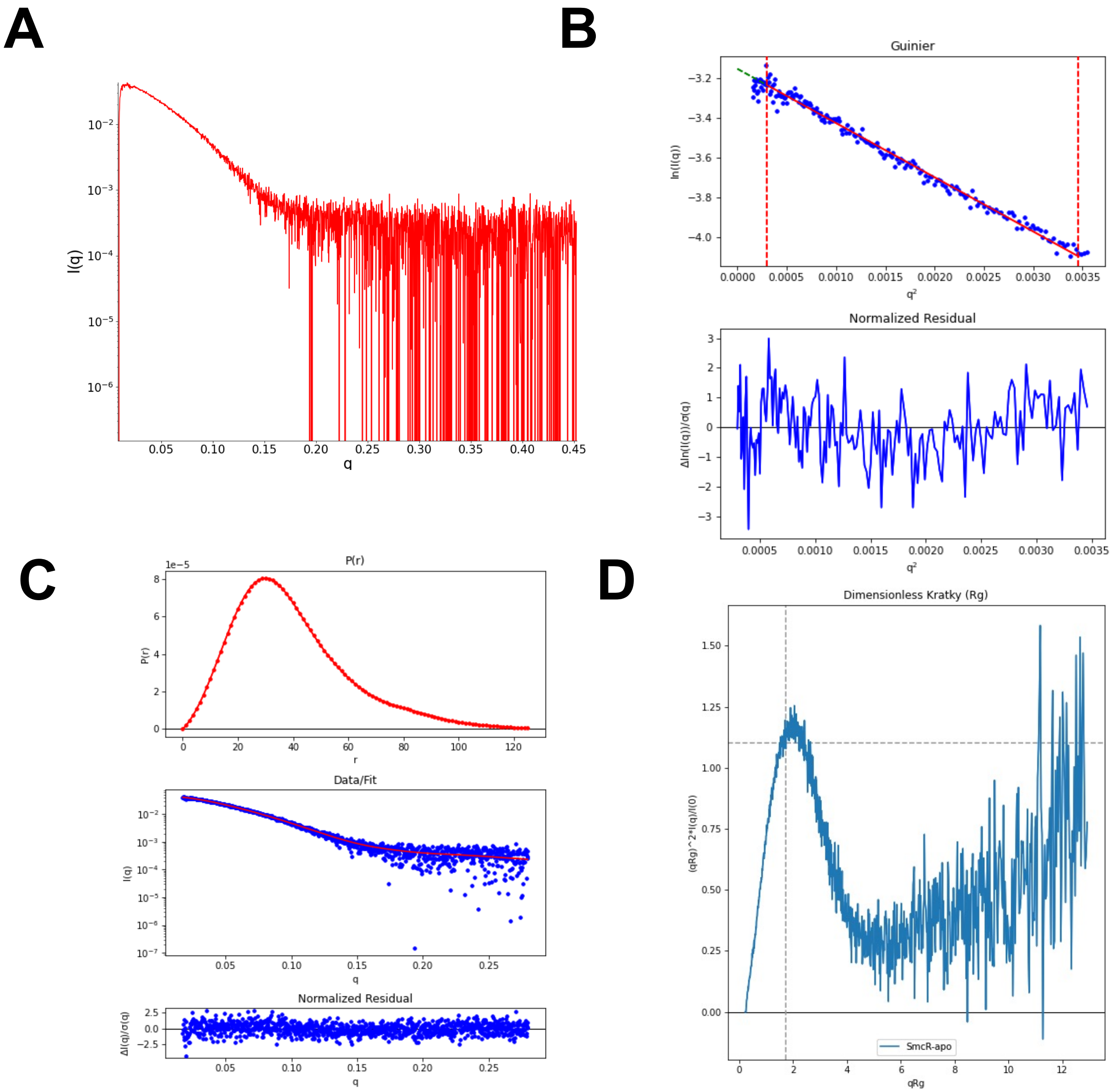

Figure S6. SAXS summary plots for SmcR-apo*.* (A) Scattering intensity profile, I(q) as a function of the scattering vector (q) on a log-lin scale. (B) Guinier plot (top panel) and normalized residuals (bottom panel). (C) The pair-distance distribution function P(r) (top panel). The scattering intensity profile calculated from the P(r) function (red) overlaid with the scattering intensity profile from the experimental data (blue, middle panel). (D) Dimensionless Kratky plot. Dashed lines show where a globular system would peak.

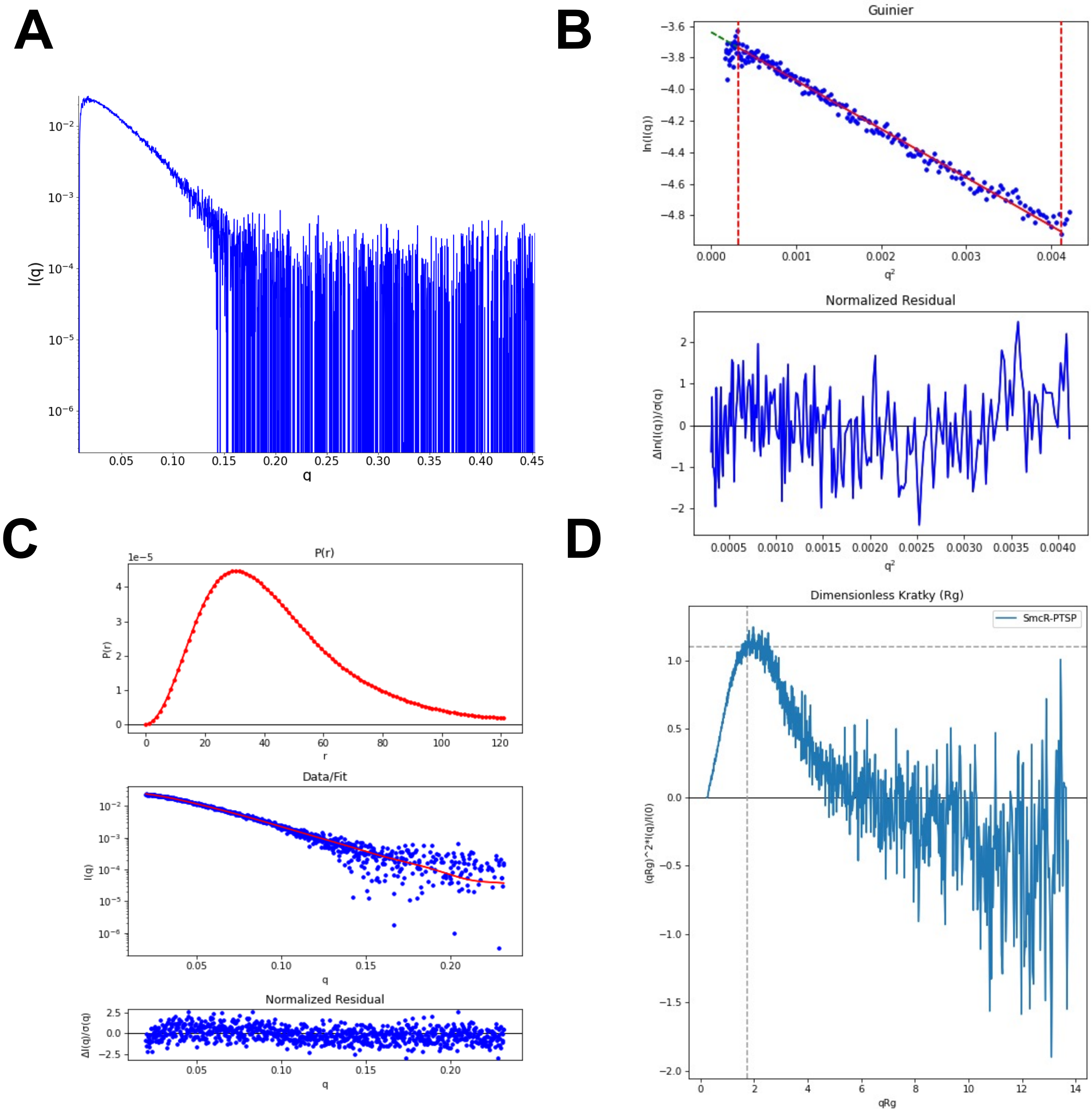

Figure S7. SAXS summary plots for SmcR-PTSP*.* (A) Scattering intensity profile, I(q) as a function of the scattering vector (q) on a log-lin scale. (B) Guinier plot (top panel) and normalized residuals (bottom panel). (C) The pair-distance distribution function P(r) (top panel). The scattering intensity profile calculated from the P(r) function (red) overlaid with the scattering intensity profile from the experimental data (blue, middle panel). (D) Dimensionless Kratky plot. Dashed lines show where a globular system would peak.

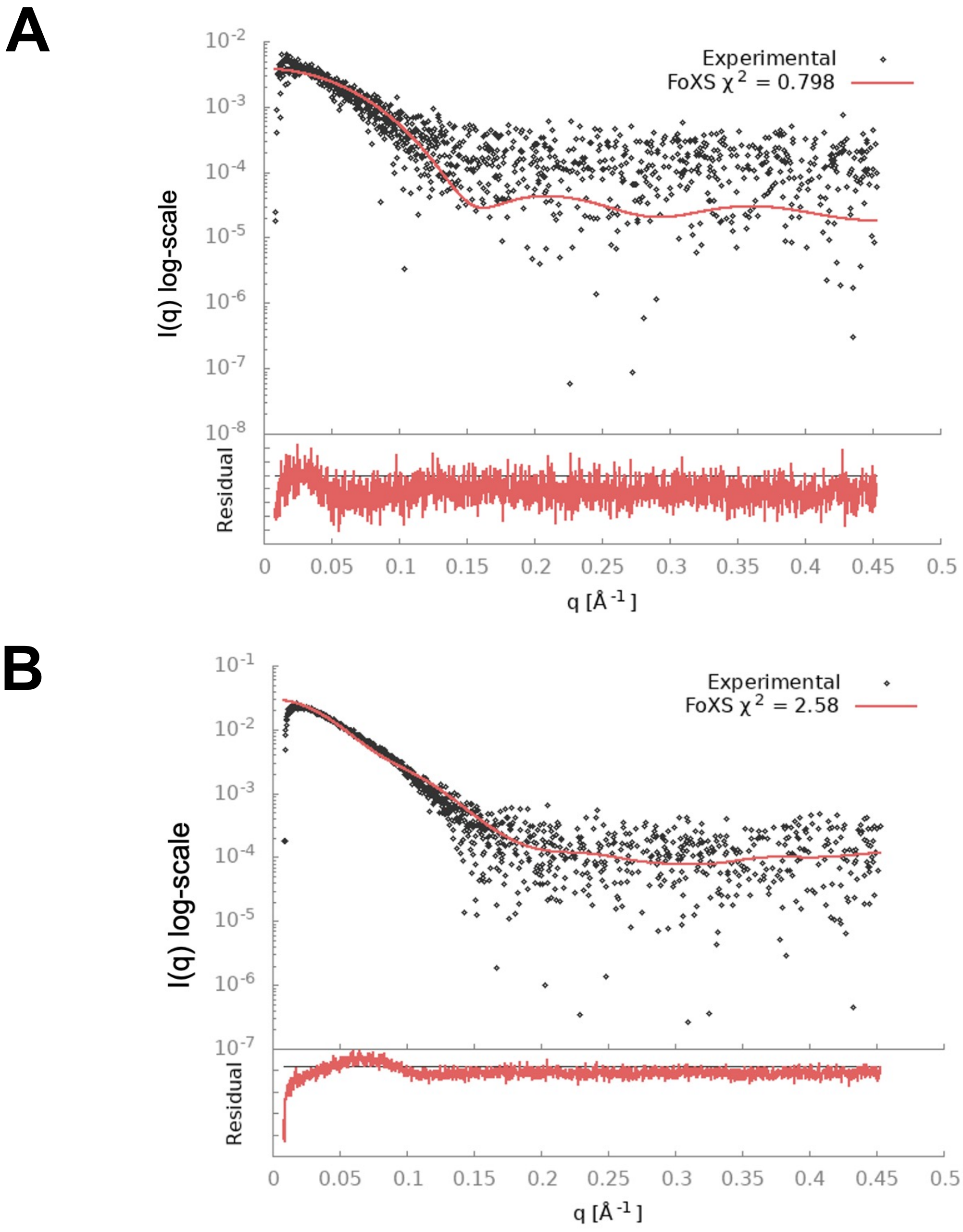

Figure S8. Single-state modeling of SmcR proteins based on experimental SAXS profiles*.* (A) The SmcR-apo SAXS experimental data (black circles) was fit to the theoretical profile derived from the high-resolution X-ray structure (PDB: 6WAE) (red line). (B) The SmcR-PTSP SAXS experimental data (black circles) was fit to the theoretical profile derived from the high-resolution X-ray structure (PDB: 6WAE) (red line). Single-state modeling was performed using the FoXS server (https://modbase.compbio.ucsf.edu/foxs/) (Schneidman-Duhovny et al., 2013; Schneidman-Duhovny et al., 2016).

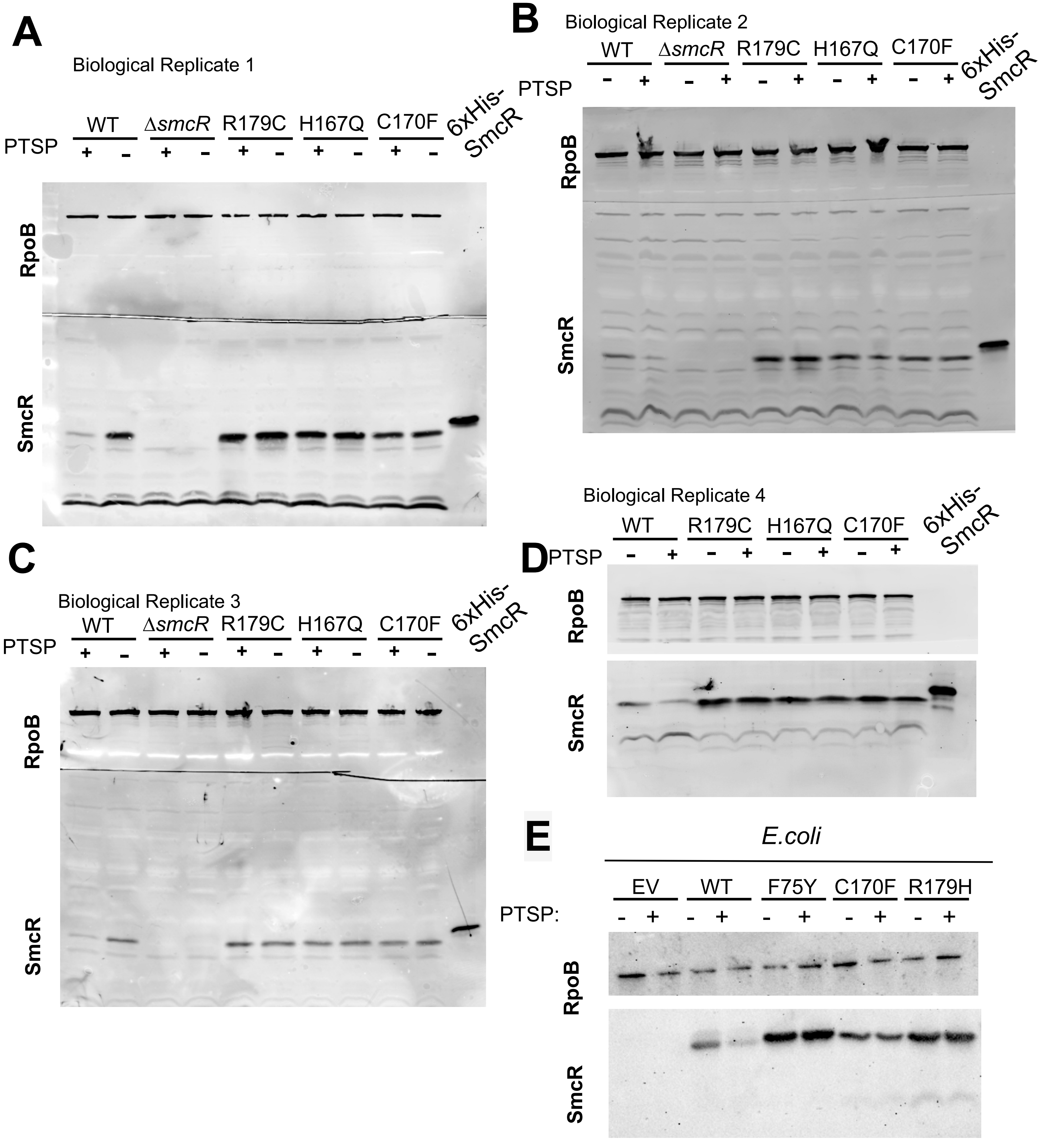

Figure S9. PTSP increases proteolytic degradation of SmcR *in vivo*. (A-D) Western blot analysis of SmcR protein levels in cell lysates from *V. vulnificus* strains wild-type, *smcR* R179C, H167Q and C170F with 25 μM PTSP (+) or an equivalent volume of DMSO solvent (-). Anti-RpoB antibodies were used as a loading control. Each panel is one biological replicate. Uncropped gels are shown. (E) Western blot analysis of SmcR protein levels in cell lysates from *E.coli* strains expressing wild-type *smcR*, *smcR* F75Y, C170F, R179H, or containing an empty vector (EV) treated with 25 μM PTSP (+) or an equivalent concentration of DMSO solvent (-). Anti-RpoB antibodies were used as a loading control.

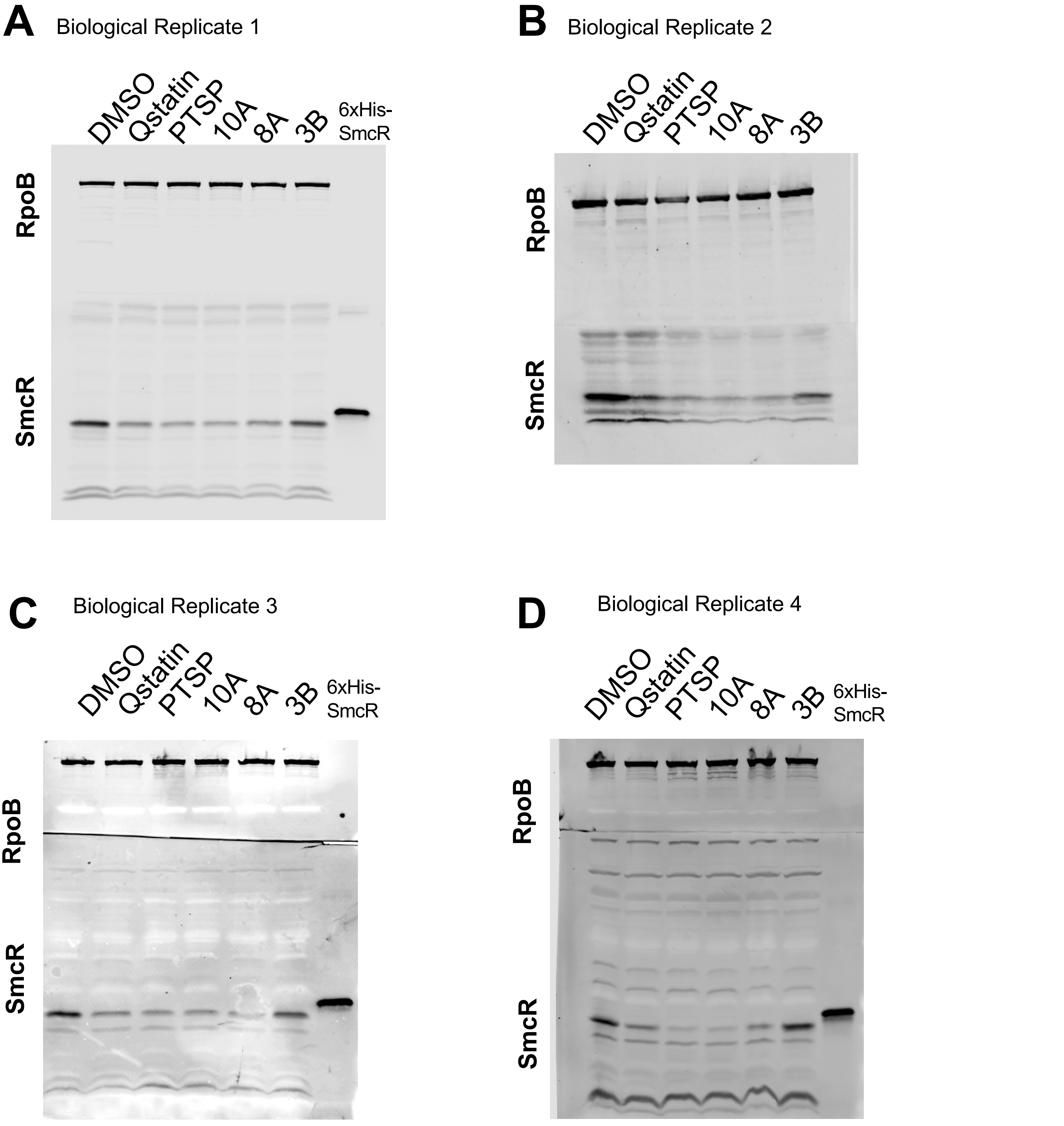

Figure S10. (A-D) Western blot analysis of SmcR protein levels in cell lysates from wild-type *V. vulnificus* treated with 25 μM of each thiophenesulfonamide or an equivalent volume of DMSO solvent. Anti-RpoB antibodies were used as a loading control. Each panel is one biological replicate. Uncropped gels are shown.

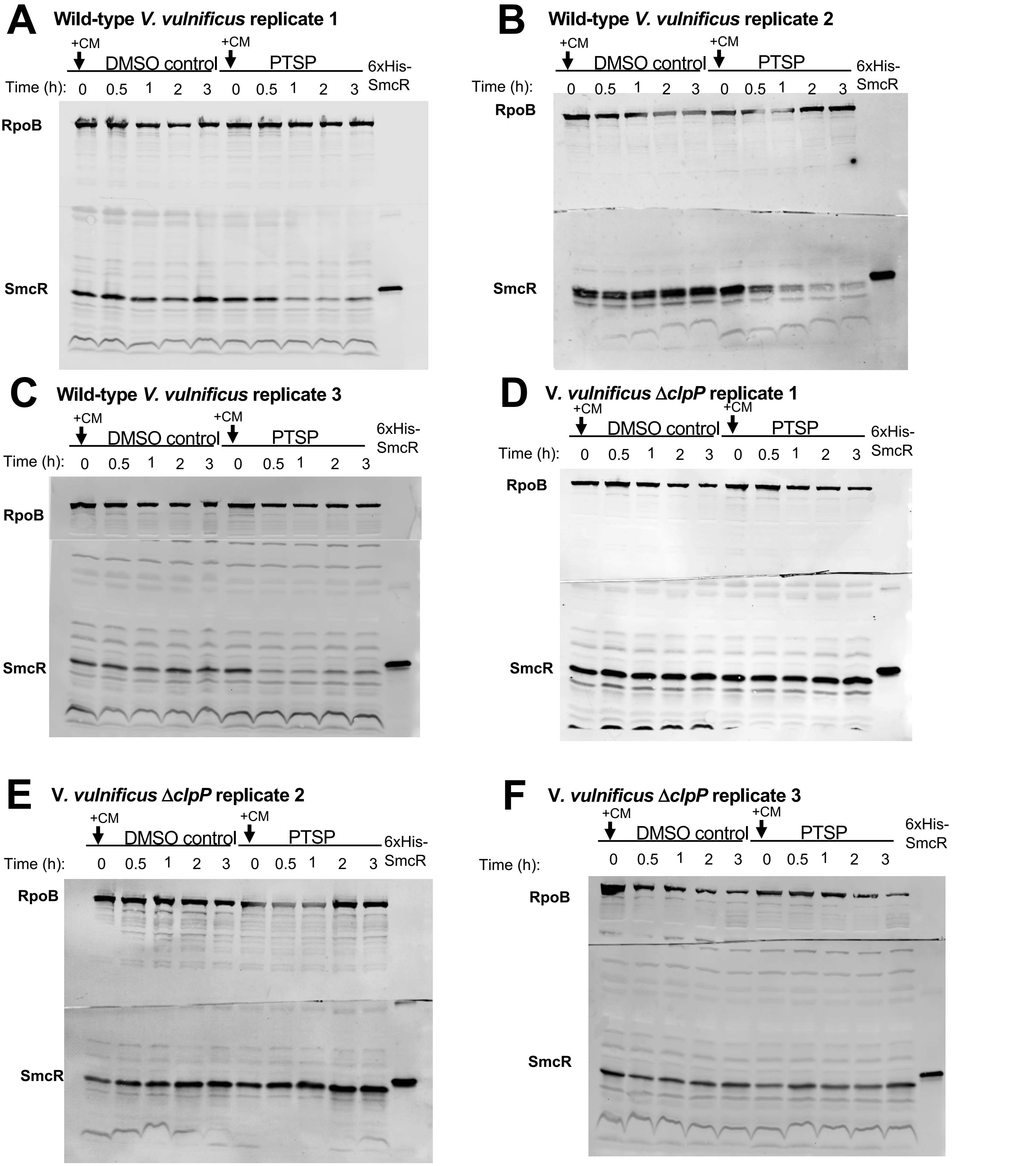

Figure S11. Time-course of SmcR protein stability following translation inhibition. (A-F) Western blot analysis of cell lysates with anti-LuxR antibodies measuring SmcR from *V. vulnificus,* wild-type (A-C) or Δ*clpP* (D-F). Cells were either treated with 25 μM PTSP or an equivalent concentration of DMSO solvent and grown to mid-log phase (OD_{600} = 0.5-0.8). Translation was halted by addition of 10 μg/ml of chloramphenicol (CM) at time 0, and pellets were harvested at 0, 30, 60, 120, and 180 mins. Anti-RpoB antibodies were used as a loading control. Each panel is one biological replicate. Uncropped gels are shown.

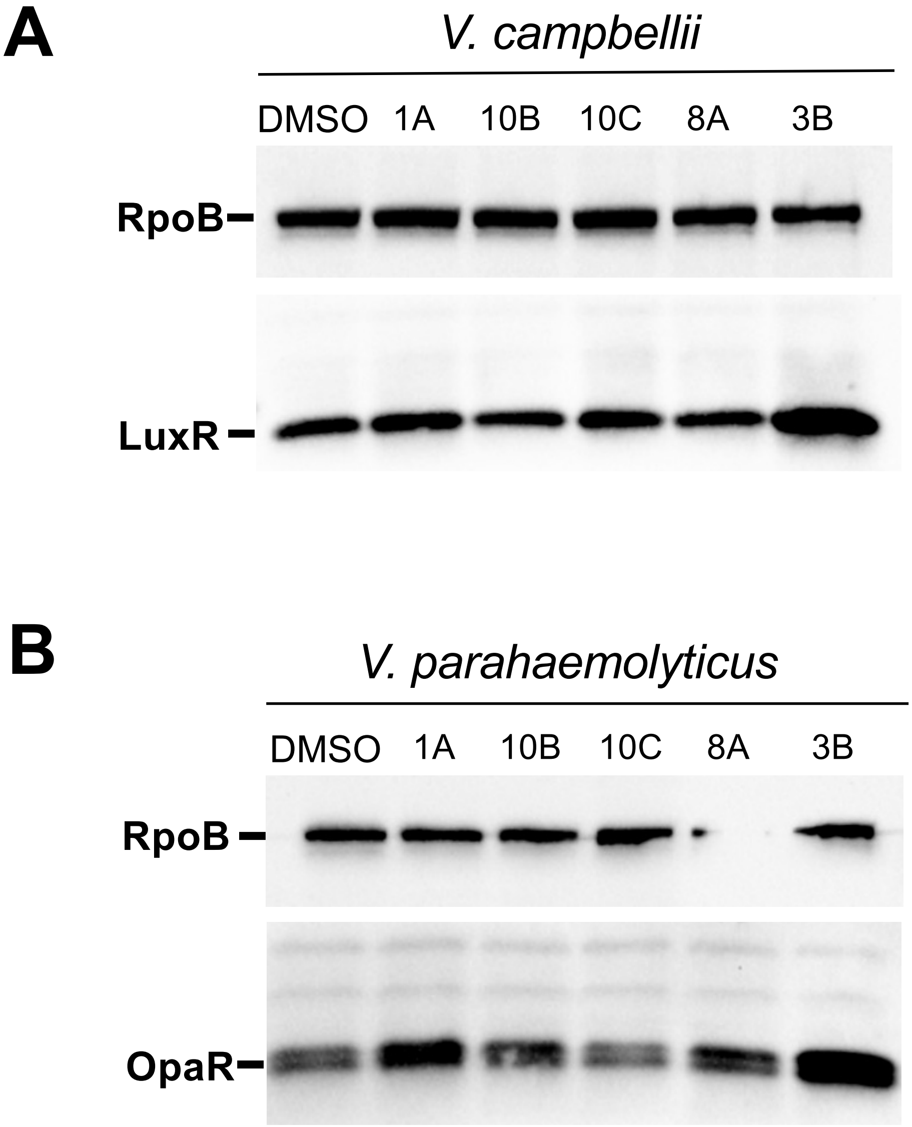

Figure S12. Thiophenesulfonamides cause proteolytic degradation of LuxR/HapR proteins in other *Vibrio* species. (A, B) Western blot analysis of cell lysates with anti-LuxR antibodies measuring LuxR from *V. campbellii* and OpaR from *V. parahaemolyticus.* Cells were treated with 25 μM of each thiophenesulfonamide indicated or an equivalent concentration of DMSO solvent (-). Anti-RpoB antibodies were used as a loading control. Each panel is a representative of triplicate biological assays.

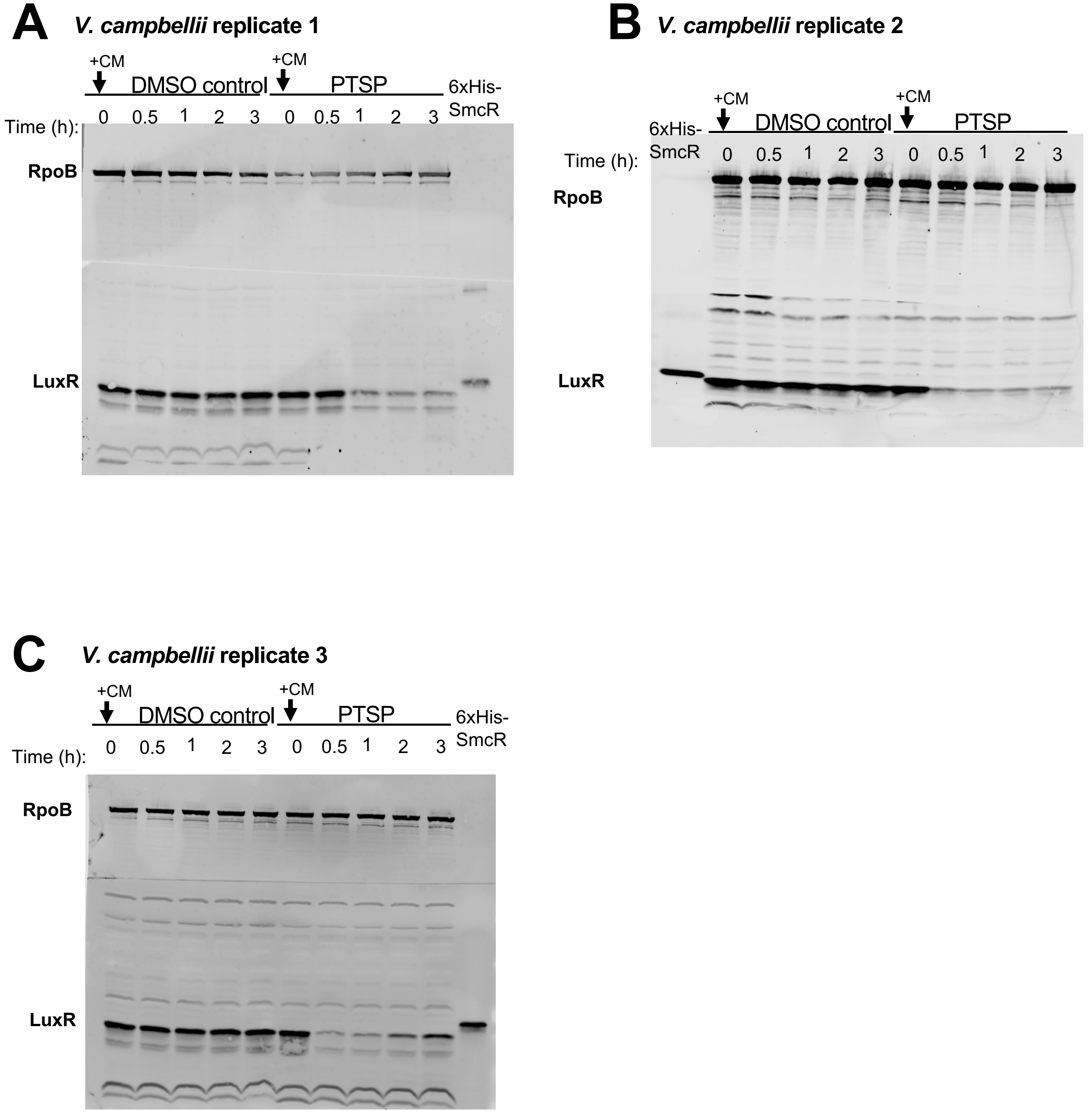

Figure S13. Time-course of LuxR protein stability following translation inhibition. (A-C) Western blot analysis of cell lysates with anti-LuxR antibodies measuring LuxR from *V. campbellii.* Cells were either treated with 25 μM PTSP or an equivalent concentration of DMSO solvent and grown to mid-log phase (OD_{600} = 0.5-0.8). Translation was halted by addition of 10 μg/ml of chloramphenicol (CM) at time 0, and pellets were harvested at 0, 30, 60, 120, and 180 mins. Anti-RpoB antibodies were used as a loading control. Each panel is one biological replicate. Uncropped gels are shown.

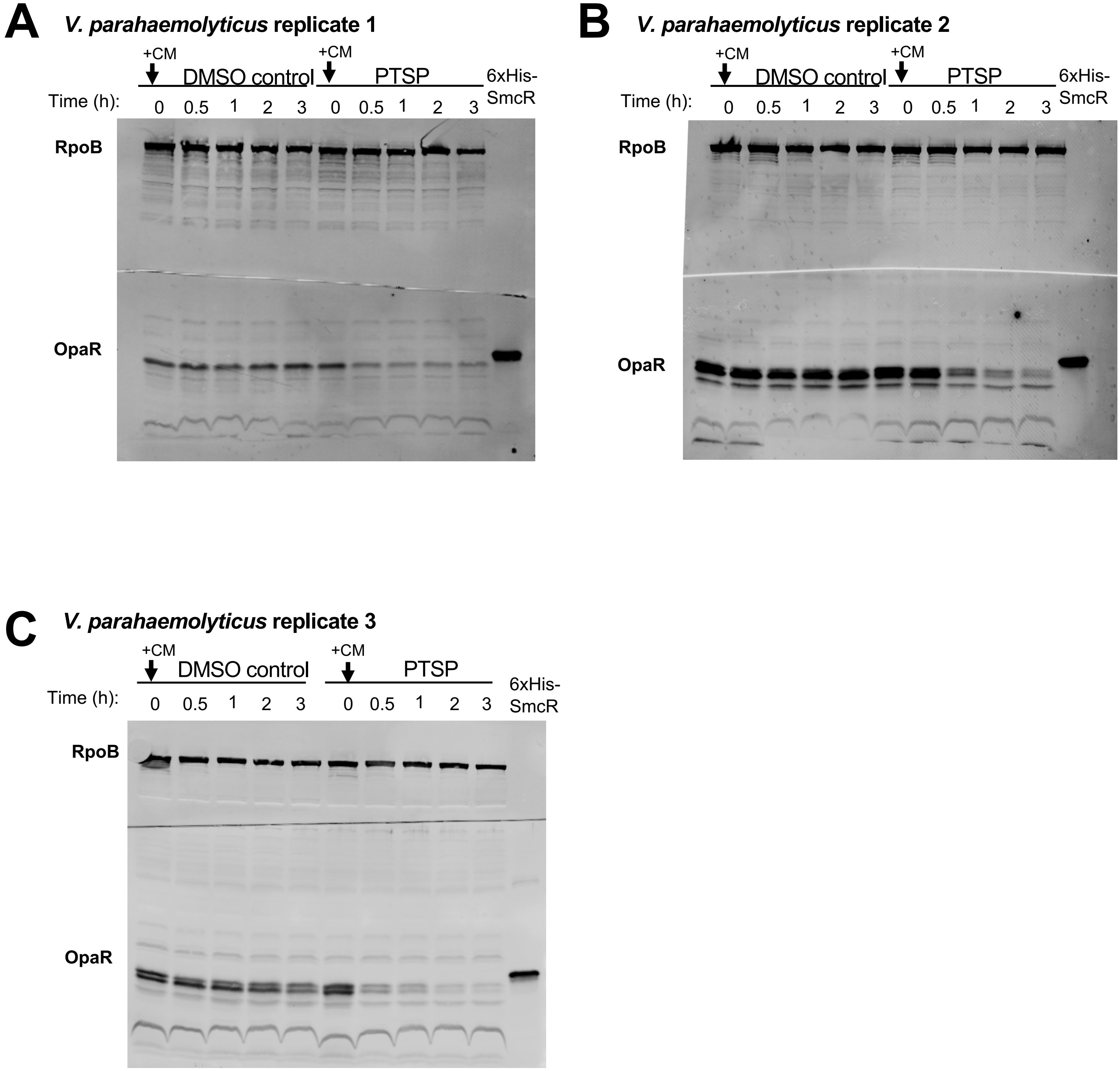

Figure S14. Time-course of OpaR protein stability following translation inhibition. (A-C) Western blot analysis of cell lysates with anti-LuxR antibodies measuring OpaR from *V. parahaemolyticus.* Cells were either treated with 25 μM PTSP or an equivalent concentration of DMSO solvent and grown to mid-log phase (OD_{600} = 0.5-0.8). Translation was halted by addition of 10 μg/ml of chloramphenicol (CM) at time 0, and pellets were harvested at 0, 30, 60, 120, and 180 mins. Anti-RpoB antibodies were used as a loading control. Each panel is one biological replicate. Uncropped gels are shown.

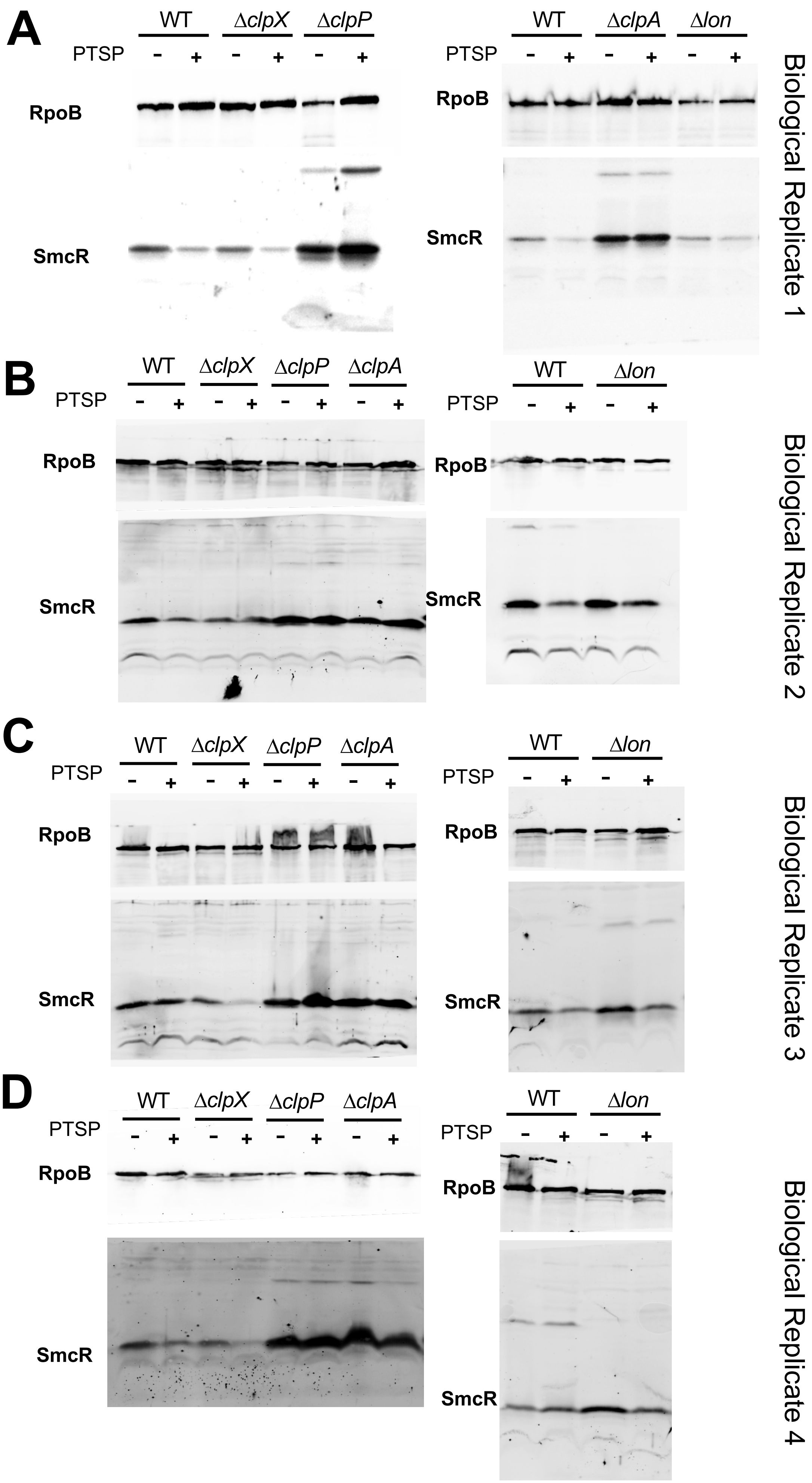

Figure S15. ClpAP proteolysis of SmcR is blocked by substitutions that block inhibitor binding. (A-D) Western blot analysis of cell lysates measuring SmcR from *V. vulnificus* strain MO6-24/0 wild-type, Δ*clpX*, Δ*clpP*, or Δ*clpA*, or Δ*lon*. Cells were treated with 25 μM of PTSP or an equivalent concentration of DMSO solvent (-). Anti-RpoB antibodies were used as a loading control. Each panel is one biological replicate. Uncropped gels are shown.

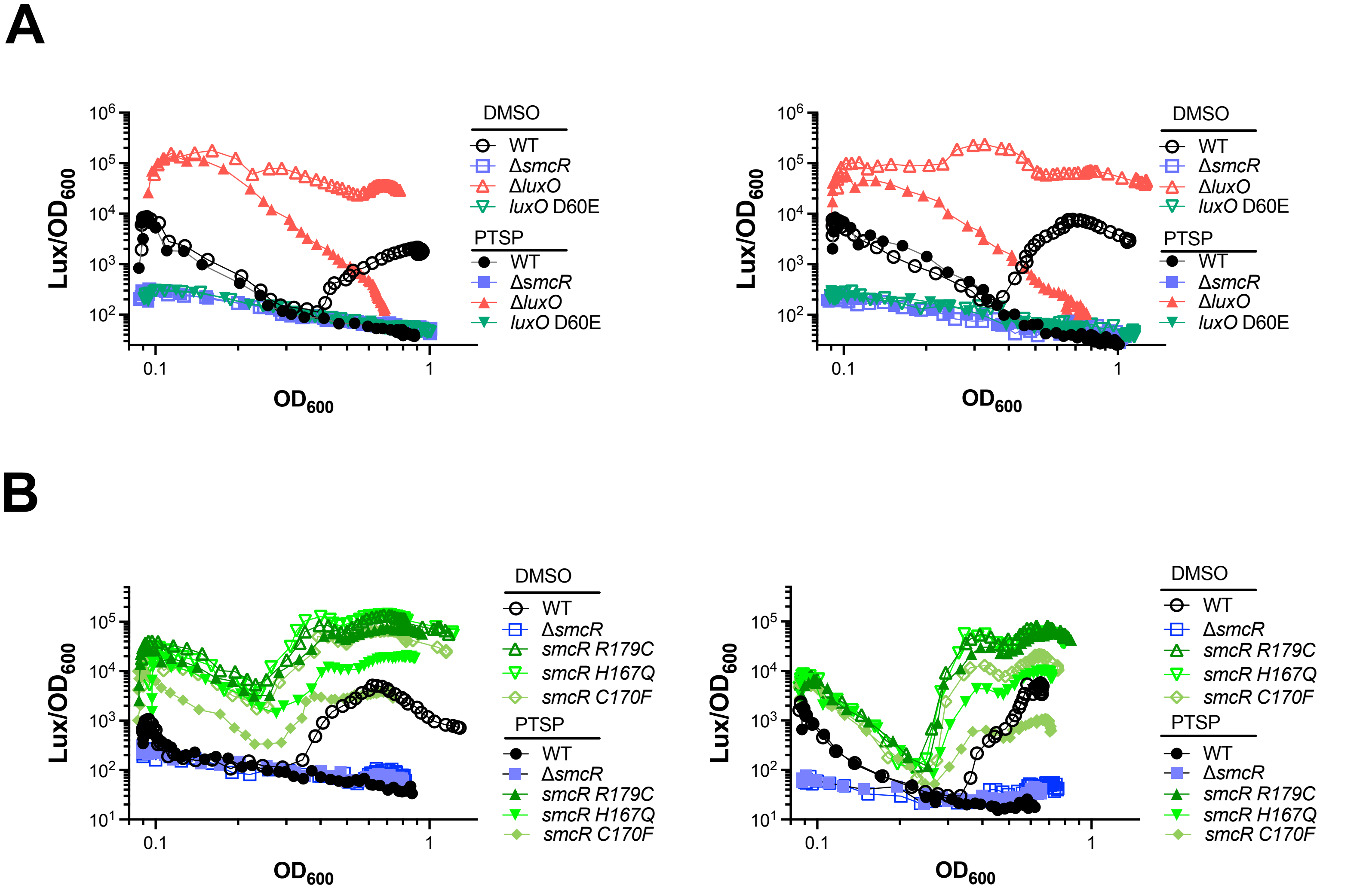

Figure S16. ClpAP proteolysis of SmcR is blocked by substitutions that block inhibitor binding. (A, B) Bioluminescence assays are plotted (Lux/OD_{600}) for *V. vulnificus* strains ATCC 27562 and isogenic mutants. Strains were treated with 25 µM PTSP (+) or an equivalent volume of DMSO solvent (-). Each panel shows two biological replicates; the third biological replicate is in the main figure.

Video S1. X-ray crystal structure of SmcR bound to PTSP (PDB: 8W39).

Table S1. Crystallographic statistics. Values in parentheses represent the highest resolution bin.

Space group	P21 21 21
Unit cell dimensions (Å)	58.01, 64.65, 138.73, 90, 90, 90
Resolution (Å)	47.29-2.10 (2.10-2.18)
Total reflections	208777 (17499)
Unique reflections	31260 (2538)
Completeness (%)	99.3 (99.0)
R_{merge}	0.125 (0.217)
Clash score	6.51
<I>/<sigma>	15.1 (7.0)
Total atoms	1409
R_{work} (%)	26.1 (36.3)
R_{free} (%)	29.1 (36.0)
Wilson B-factor	22.0
**Number of non-hydrogen atoms**	2898
Macromolecules	2796
Ligands	38
Solvent	64
**R.m.s. deviation from ideality**	
Bond lengths (Å)	0.010
Bond angles (°)	1.71
Dihedral angles (°)	16.8
**Phi-Psi values (Ramachandran)**	
Favored (%)	96.19
Allowed (%)	3.23
Outliers (%)	0.59
Rotatmer outliers	4.61%
**Average B-factor**	54.50
Macromolecules	54.92
Ligands	36.01
Solvent	47.07

Table S2. SAXS data collection and modeling parameters.

	SmcR-apo	SmcR-PTSP
**Data-collection parameters**		
Wavelength (Å)	1.094	1.094
Camera length (m)	1.755	1.755
q-measurement range	0.0079-0.45	0.0079-0.45
Concentration (mg/mL)	2.01	1.24
**Structural parameters**		
I(0) from Guinier	0.04 ± 0.00145	0.03 ± 0.00132
R_g (Å) from Guinier (± SE)	28.58 ± 0.1	30.35 ± 0.13
R_g (Å) from GNOM (± SE)	29.57 ± 0.57	31.58 ± 0.54
I(0) from GNOM	0.04 ± 0.00345	0.03 ± 0.00331
D_{max} (Å)	125	121
MW^{vp} (kDa)	55.1	93.3

R_g: Radius of gyration. I(0): scattering intensity at zero scattering angle. D_{max}: Maximum particle diameter. MW: molecular mass.

Table S3. Bacterial strains used in this study.

Name	Genotype/Notes	Reference
***E. coli* strains**		
JV-Ec1008	*smcR* F75Y in pMMB67EH-kanR with pJV064	This study
JV-Ec1011	*smcR* C170F in pMMB67EH-kanR with pJV064	This study
JDN114	pMMB67EH-kanR empty vector with pJV064	(1)
JDN115	*smcR* in pMMB67EH-kanR with pJV064	(1)
TAR-ec001	*smcR* F166C in pMMB67EH-kanR with pJV064	This study
TAR-ec005	*smcR* R179H in pMMB67EH-kanR with pJV064	This study
TAR-ec006	*smcR* F78L in pMMB67EH-kanR with pJV064	This study
TAR-ec007	*smcR* H167Q in pMMB67EH-kanR with pJV064	This study
TAR-ec010	*smcR* R179C in pMMB67EH-kanR with pJV064	This study
TAR-ec012	*smcR* H167R in pMMB67EH-kanR with pJV064	This study
TAR-ec027	*smcR* Q137P in pMMB67EH-kanR with pJV064	This study
TAR-ec040	*smcR* C170Y in pMMB67EH-kanR with pJV064	This study
TAR-ec041	*smcR* I96H in pMMB67EH-kanR with pJV064	This study
TAR-ec042	*smcR* M100L in pMMB67EH-kanR with pJV064	This study
S17-1λpir	Conjugation strain, λ-pir, recA thi pro hsdR- M+ RP4: 2-Tc, Mu, TpR, SmR	(2)
DH10B	str. K-12 F– Δ(ara-leu)7697[Δ(rapA'-cra')] Δ(lac)X74[Δ('yahHmhpE)] duplication (514341-627601) [nmpCgltI] galK16 galE15 e14–(icdWT mcrA) φ80dlacZΔM15 recA1 relA1 endA1 Tn10.10 nupG rpsL150 (StrR) rph+ spoT1 Δ(mrr-hsdRMS- mcrBC) λ– Missense (dnaA glmS glyQ lpxK mreC murA) Nonsense (chiA gatZ fhuA? yigA ygcG) Frameshift(flhC mglA fruB)	Life Technologies
BL21(DE3)	*E. coli* str. B F– ompT gal dcm lon hsdSB(rB–mB–) λ(DE3 [lacI lacUV5-T7p07 ind1 sam7 nin5]) [malB+]K-12(λS)	NEB
***V. vulnificus* strains**		

ATCC 27562	*wild-type*	ATCC
CAS-vv015	ATCC 27562 *ΔsmcR::Spec*^{R}, pMMB67EH-tfoX-kanR	(3)
CAS-vv022	ATCC 27562 *smcR-Km*^{R}*,* pCS32	(3)
CAS-vv026	ATCC 27562 *smcR-Tm*^{R}, pCS32	This study
TAR-vv001	ATCC 27562 *smcR R179C-Tm*^{R}, pCS32	This study
TAR-vv002	ATCC 27562 *smcR H167Q-Tm*^{R,} pCS32	This study
TAR-vv004	ATCC 27562 *smcR C170F- Tm*^{R,} pCS32	This study
MO6-24/O	*wild-type*	(4)
KL321	MO6-24/O, *ΔclpP*	(4)
KL322	MO6-24/O, *ΔclpA, Km*^{R}	(4)
KL421	MO6-24/O, *Δlon, Km*^{R}	(4)
SM801	MO6-24/O, *ΔclpX, Km*^{R}	(4)
TAR-vv101	ATCC 27562, pCS38-Gent^{R}	This study
TAR-vv103	ATCC 27562, *ΔsmcR::Spec*^{R}, pCS38-Gent^{R}	This study
TAR-vv105	ATCC 27562 *smcR R179C-Tm*^{R,} pCS38-Gent^{R}	This study
TAR-vv107	ATCC 27562 *smcR H167Q-Tm*^{R,}, pCS38-Gent^{R}	This study
TAR-vv109	ATCC 27562 *smcR C170F-Tm*^{R,}, pCS38-Gent^{R}	This study
TAR-vv125	ATCC 27562, Δ*clpP::Tm*^{R}	This study
FA-021	ATCC 27562, Δ*luxO::Tm*^{R}, pCS38-Gent^{R}	This study
FA-055	ATCC 27562, *luxO* D60E, pCS38-Gent^{R}	This study
***V. campbellii* strains**		
BB120	*wild-type*	(5)
***V. parahemolyticus* strains**		
RIMD 2210633	*wild-type*	ATCC

Table S4. Oligonucleotides used in this study.

Name	Direction	Sequence	Description
TAR001	F	GACAATTAATCATCGGCTCGTATAATG	pJN22 sequencing, F
TAR002	R	GCGTTCTGATTTAATCTGTATCAGGC	pJN22 sequencing, R
TAR004	F	CAGAGAAACGCTAACTCAGGTACGCGGATG GAGGAGGGCCCTAGGAC	Primer to amplify SmcR insert from WT ATCC 27562
TAR007	R	CTCATCCGCCAAAACAGCCAAGCTCTATTC GTGTTCGCGTTTATAGATG	Primer to amplify SmcR insert from WT ATCC 27562
TAR010	F2	TCGCTGTTTGTTCAAGCAAACTGCACCAACA ATACTGCAGAGCTA	F2 primer SmcR R179C
TAR011	R1	TAGCTCTGCAGTATTGTTGGTGCAGTTTGCT TGAACAAACAGCGA	R1 primer SmcR R179C
TAR012	R	CTCAGGTCGACGGATCCCCGGAAT	R sequencing primer to check SmcR mutations in CAS-vv022
TAR015	F2	GAAGATTTGGCAAACCTGTTCCAAGGCATTT GTTACTCGCTGTTT	F2 primer SmcR H167Q
TAR016	R1	AAACAGCGAGTAACAAATGCCTTGGAACA GGTTTGCCAAATCTTC	R1 primer SmcR H167Q
TAR044	F1	AGCGTCGAACTCCACCCCAGTTGG	up strand F1, clpP deletion in WT vv026
TAR045	R1	GTCGACGGATCCCCGGAATACTCATCGTAG TGCATAATTTGCCTGC	clpP up strand R1 with homology to ABD123, clpP deletion in WT vv026

TAR046	F2	GAAGCAGCTCCAGCCTACATAAGCTGGTTG GTTCATTAGCTCG	clpP down strand F2 with homology to ABD124, clpP deletion in WT vv026
TAR047	R2	GAACTTCGCAGGATCGATGCCTGG	clpP down strand R2, clpP deletion in WT vv026
TAR048	R	GAACTAAACGACGAGTTCGTT	delta clpP detection primer reverse
JCV1311	F2	ATTTGGCAAACCTGTTCCACGGCATTTTCTA CTCGCTGTTTGTTCAAGCAAACCGC	F2 primer SmcR C170F
JCV1312	R1	GCGGTTTGCTTGAACAAACAGCGAGTAGAA AATGCCGTGGAACAGGTTTGCCAAAT	R1 primer SmcR C170F
CAS107	F1	CGTCTCAGGCAACGCAGACAGTG	pMMB amplify backbone, F
CAS110	R2	GGAAGCTCAAGCAACGACTAGTG	pMMB amplify backbone, R
TAR029	F1	AATCGTCTCAGGCAACGCAGACAGTGTAC	Redesigned CAS107
TAR030	R2	AGCGGAAGCTCAAGCAACGACTAGTGATT	Redesigned CAS110
ABD123	R	ATTCCGGGGATCCGTCGAC	Forward primer to amplify antibiotic resistance cassettes
ABD124	F	TGTAGGCTGGAGCTGCTTC	Reverse primer to amplify antibiotic resistance cassettes
JCV 149	F	CGTGTCGCTCAAGGCGCACTCCCGT	pJN22 amplify backbone, F
JDN104	R	ATCGAGTTGCGGCCGCTTGTTGTTACCTTA GCAGGGTG	pJN22 amplify backbone, R
CAS536	R	CAACCAACAGCCATTGGTTATAGG	R to detect in TmR cassette for seq before TmR

Table S5. Plasmids used in this study.

Name	Description	Reference
pJN08	6x-his-smcR in pET28b	(6)
pJN22	P_{tac}-smcR (V. vulnificus ATCC 27562), in pMMB67EH-kanR	(6)
pJV064	P_{05222}-mCherry, P_{luxC}-gfp; p15a origin, CM^R	(7)
pCS38	*luxCDABE* region, in pMMB67EH-kanR	(8)
pMMB67EH-kanR	Vector control; kanR	(3)
pTR003	*His-smcR R179C*, pET28a.parent vector	This study, TWIST

References

1. J. D. Newman, *et al.*, Amino acid divergence in the ligand-binding pocket of Vibrio LuxR/HapR proteins determines the efficacy of thiophenesulfonamide inhibitors. *Mol Microbiol* **116**, 1173–1188 (2021).
2. V. De Lorenzo, K. N. Timmis, “386 GENETICS AND REGULATION [31] [3 1] Analysis and Construction of Stable Phenotypes in Gram-Negative Bacteria with Tn5-and Tnl0-Derived Minitransposons” (1994).
3. C. A. Simpson, R. Podicheti, D. B. Rusch, A. B. Dalia, J. C. Van Kessel, Diversity in Natural Transformation Frequencies and Regulation across Vibrio Species. (2019). https://doi.org/10.1128/mBio.
4. K. J. Lee, Y. C. Jung, S. J. Park, K. H. Lee, Role of heat shock proteases in quorum-sensing-mediated regulation of biofilm formation by Vibrio species. *mBio* **9** (2018).
5. B. L. Bassler, E. P. Greenberg, A. M. Stevens, “Cross-Species Induction of Luminescence in the Quorum-Sensing Bacterium Vibrio harveyi” (1997).

6. J. D. Newman, *et al.*, The DNA binding domain of the Vibrio vulnificus SmcR transcription factor is flexible and binds diverse DNA sequences. *Nucleic Acids Res* **49**, 5967–5984 (2021).
7. J. C. van Kessel, L. E. Ulrich, I. B. Zhulin, B. L. Bassler, Analysis of activator and repressor functions reveals the requirements for transcriptional control by LuxR, the master regulator of quorum sensing in Vibrio harveyi. *mBio* **4** (2013).
